# Identification of rare disease genes as drivers of common diseases through tissue-specific gene regulatory networks

**DOI:** 10.1038/s41598-024-80670-1

**Published:** 2024-12-04

**Authors:** Olivier B. Bakker, Annique Claringbould, Harm-Jan Westra, Henry Wiersma, Floranne Boulogne, Urmo Võsa, Carlos G. Urzúa-Traslaviña, Sophie Mulcahy Symmons, Mahmoud M. M. Zidan, Marie C. Sadler, Zoltán Kutalik, Iris H. Jonkers, Lude Franke, Patrick Deelen

**Affiliations:** 1 Department of Genetics, University Medical Center Groningen, University of Groningen, Groningen, The Netherlands; 2 Structural and Computational Biology Unit, EMBL, Heidelberg, Germany; 3 Internal Medicine, Erasmus Medical Centre Rotterdam, Rotterdam, The Netherlands; 4 Oncode Institute, Utrecht, The Netherlands; 5 Estonian Genome Centre, Institute of Genomics, University of Tartu, Tartu, Estonia; 6 University Center for Primary Care and Public Health, 1010 Lausanne, Switzerland; 7 Department of Computational Biology, University of Lausanne, Lausanne, Switzerland; 8 Swiss Institute of Bioinformatics, Lausanne, Switzerland

**Keywords:** Gene expression, Population genetics

## Abstract

Genetic variants identified through genome-wide association studies (GWAS) are typically non-coding, exerting small regulatory effects on downstream genes. However, which downstream genes are ultimately impacted and how they confer risk remains mostly unclear. By contrast, variants that cause rare Mendelian diseases are often coding and have a more direct impact on disease development. Here we demonstrate that common and rare genetic diseases can be linked by studying how common disease-associated variants influence gene co-expression in 57 different tissues and cell types. We implemented this method in a framework called Downstreamer and applied it to 88 GWAS traits. We find that predicted downstream “genes” are enriched with Mendelian disease genes, e.g. key genes for height are enriched for genes that cause skeletal abnormalities and Ehlers–Danlos syndromes. 78% of these key genes are located outside of GWAS loci, suggesting that they result from complex *trans* regulation rather than being impacted by disease-associated variants in *cis*. Based on our findings, we discuss the challenges in reconstructing gene regulatory networks and provide a roadmap to improve the identification of these highly connected genes linked to common traits and diseases.

## Introduction

Genetic variation plays a major role in the development of both common and rare diseases, yet the genetic architectures of these two types of disease are usually considered to be quite different. Rare genetic disorders are thought to be primarily caused by a single genetic variant that has a large effect on disease risk, most often through effects on protein coding. In contrast, the genetic risk for common diseases is modulated by many, mostly non-coding, variants that individually exert small effects, as identified through genome-wide association studies (GWASs). However, identification of the causal variants and genes affected by GWAS loci remains challenging, due in part to unknown mechanisms of action, linkage disequilibrium (LD) and small effect sizes^1,2^.

Despite the differences between rare and complex diseases, GWAS loci have been shown to be enriched for genes that can cause related rare diseases when damaged^3,4^. For instance, common variants associated to PR interval, a measurement of heart function, have been found within the *MYH6* gene^5^ known to harbour mutations in individuals with familial dilated cardiomyopathy^6^. Moreover, eQTL studies have found examples of rare disease genes that are affected by distal common variants in *trans*, such as the immunodeficiency gene *ISG15*, which is affected by multiple variants associated with systemic lupus erythematosus^7^. These results indicate that both common and rare diseases can result from damage to or altered regulation of the same genes, suggesting that the same biological pathways underlie these conditions^4^. However, it is not fully known to what extent specific genes and pathways are shared between rare and common diseases.

Recent years have seen the development of multiple pathway-enrichment methods that can identify which biological pathways are enriched in common diseases^8–10^ and highlight their most likely cellular context(s)^11–14^. In addition, several methods can prioritise individual genes within GWAS susceptibility loci by studying how they are functionally related to genes in other susceptibility loci^8,15–17^. However, these methods confine themselves to genes in GWAS loci, potentially missing relevant *trans-*regulated up- or downstream effects. More recent approaches do take *trans* regulation into account, operating either through network propagation or a linear scoring metric combined with a network to prioritise connected genes^18–20^. Another approach to identify *trans* effects is to map expression quantitative trait loci (eQTL). In blood, this approach has successfully identified the downstream *trans* regulatory consequences of GWAS-associated variants (i.e. *trans*-eQTLs and eQTSs, where polygenic scores are linked to expression levels)^7^. To gain actionable insight into disease biology, eQTL need to be mapped in a disease-relevant context. However, given the large sample sizes required to detect such effects, the available datasets for most tissues, cell states and diseases are currently too small.

Here, rather than mapping *trans* effects directly, we build upon the ‘omnigenic model’ hypothesis. This model states that the most important disease genes in complex diseases are modulated by many different common variants through gene regulatory networks^21,22^. The omnigenic model postulates that a limited number of core genes drive diseases, while the many peripheral genes, which may contain associated variants, only contribute to disease development by modulating the activity of the core genes. Since the omnigenic model does not require that core genes map inside GWAS loci, many core genes will likely be missed by methods that prioritise genes within GWAS loci. The omnigenic model hypothesis is supported by recent work assessing RNA-levels of blood cells^23^ and molecular traits^24^, as well as a large-scale in vitro knockdown experiment^25^. However, these studies were performed in blood, limiting their conclusions to GWAS studies on blood-related traits and immunological disorders.

Building on this work, we used tissue-specific gene-modules derived from 46,410 RNA-seq samples from 57 different tissues and cell types. We created these gene-modules using an eigen decomposition of the tissue-specific expression matrices, which resulted in gene-modules describing how genes are co-expressed within each of the 57 tissues we studied. We then combined these gene-modules with GWAS summary statistics to prioritise ‘key genes’ in a framework we call Downstreamer. We found that the prioritised genes are more likely to contribute directly to disease predisposition than genes prioritised through GWAS alone, we applied Downstreamer to 88 GWASs for a wide variety of traits (Table S1). This analysis demonstrated that the prioritised genes are enriched for intolerance to loss-of-function (LoF) and missense (MiS) mutations and for Mendelian disease genes that lead to phenotypic outcomes similar to the GWAS trait. Prioritised genes that cause Mendelian disease can therefore highlight the molecular pathways driving the complex disease, and we used this criterion to determine a subset of prioritised key genes. Conversely, since we observe that the set of key genes is enriched with known rare disease genes, we hypothesise that other key genes might contain damaging alleles that could explain disease in rare disease patients who have not yet been genetically diagnosed. We have made Downstreamer freely available at: https://github.com/molgenis/systemsgenetics/wiki/Downstreamer.

## Results

### Overview of the Downstreamer framework

**Fig. 1:**
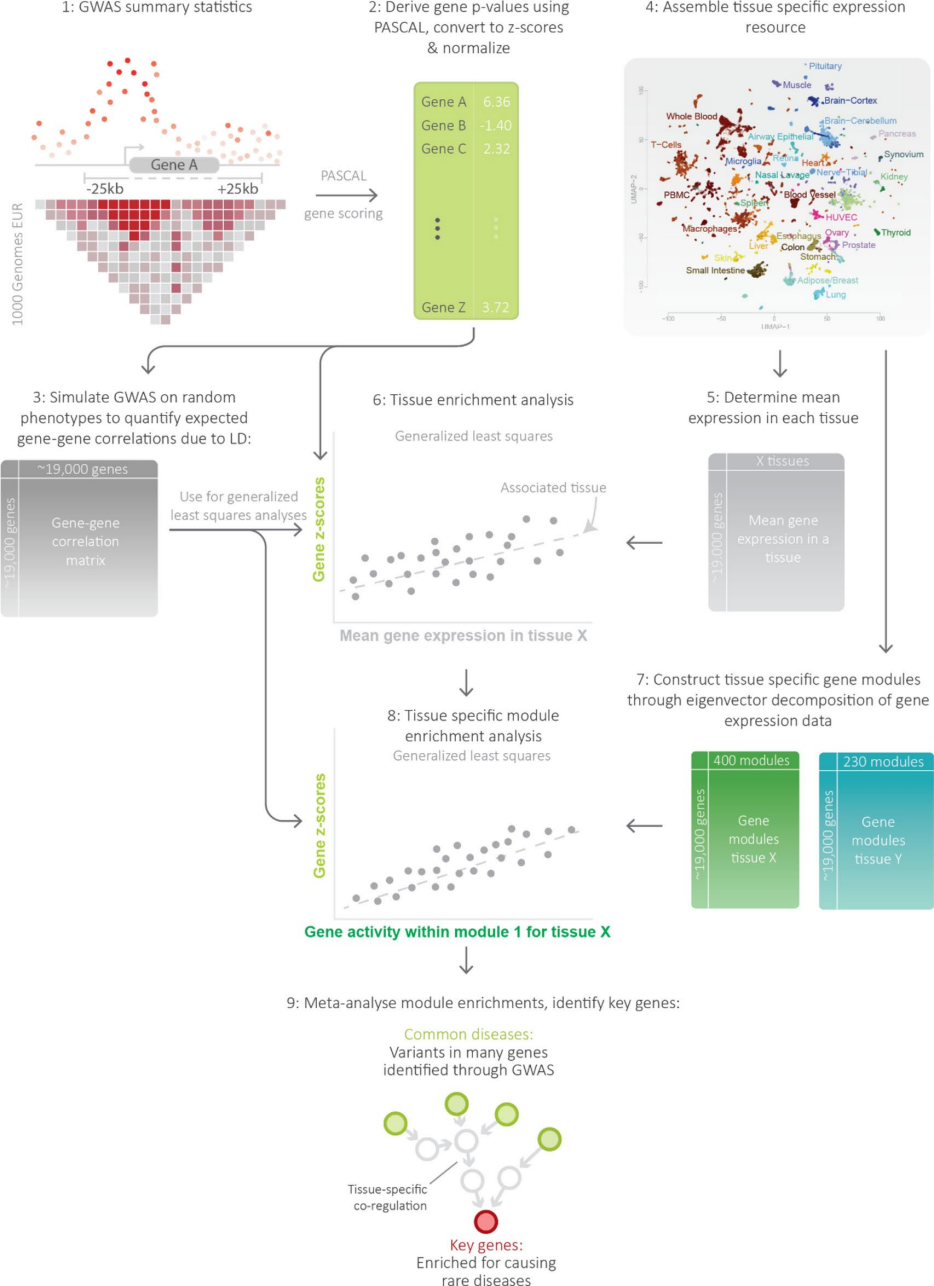
Overview of the Downstreamer approach. Starting from genome-wide association study (GWAS) summary statistics (1), individual SNP p-values were aggregated into gene-level p-values using PascalX, taking linkage disequilibrium (LD) into account (2). 10,000 random GWAS profiles were then generated to estimate the gene–gene correlation expected due to LD (3). We then utilised the Recount3 resource (4) to derive mean expression profiles for the 57 available cell types and tissues (5). We associated this profile to the GWAS profile in a generalised least squares framework that controls for the effects of LD captured in the gene–gene correlation matrix (6). This resulted in one association per GWAS profile for each of the 57 tissues. We also created tissue-specific gene-modules, which are eigenvectors calculated on the subset of genes that are expressed in the respective tissue (7). For tissues with a significant association, we calculated enrichment of tissue-specific gene-modules (8). Resulting enrichments were then meta-analysed to derive a cross-tissue relevance score for a gene in a given GWAS (9).

To enable identification of GWAS key genes, we developed the Downstreamer framework (Fig. 1). In this framework, we first converted variant-level *p*-values to gene-level z-scores using PascalX^26^. Next, we curated a resource of gene-expression data from Recount3^27^ containing 57 primary tissues (Table S2), in which we identified tissue-specific gene-modules. We associated these gene-modules to the gene-level z-scores from GWAS for the tissues that showed relatively high expression for the genes in the GWAS loci (Methods). Using the enriched gene-modules, we derived a “key gene score” for each tissue–trait pair. These tissue-specific key gene scores were then meta-analysed as a cross-tissue measure of gene relevance. We define the “key genes” for a GWAS trait as those genes that have a significant key gene score and a known association to a phenotype-matched rare disease.

### Association signals shared among polygenic traits cluster around transcription factors and cell-cycle genes

The first step in Downstreamer is to convert individual variant associations to gene p-values by aggregating associations within a 25kb window around the gene body for all protein-coding genes using the PascalX approach^26^ (Fig. 1). PascalX needs an LD reference panel to ensure that the gene *p*-values are not confounded by multiple variants tagging the same association. PascalX uses the LD information to correct the gene *p*-values for shared tagging. These gene p-values are then converted to gene-level z-scores. We note the sign of the gene-level z-scores does not reflect the sign of the GWAS effect. Using this approach, we calculated gene z-scores for 88 GWAS summary statistics reflecting a wide variety of disorders and complex traits (Table S1).

Based on this analysis, we observed that the z-scores of individual GWASs were often weakly positively correlated (Fig. S1A), especially for traits for which many loci have been identified (Fig. S1B,C). For instance, the gene-level z-scores for height correlated positively with the gene-level z-scores of all other traits. To investigate the source of this shared signal, we calculated the average gene-level association across all 88 traits while correcting for the bias that might be introduced for traits that are strongly correlated (see “Methods” Section). Here we found that 15% of the variation in the ‘average GWAS’ signal could be explained by both the extent of LD around a gene and the local gene density (Fig. S2). We next evaluated if the remaining 85% of the average signal was enriched for any biological processes. After correcting for LD and gene density, we observed that 102 of the top 500 genes are transcription factors (Odds ratio (OR): 2.95, *p*-value < 2.2 × 10^−16^). We also saw enrichments for general pathways, mainly related to transcription, RNA metabolism and cell cycle (Table S3). Additionally, genes with a higher average gene z-score showed increased LoF intolerance (R: 0.18, *p*-value = 9.70 × 10^−125^, Fig. S3). These enrichments suggest that there is a set of genes that is enriched for GWAS hits. Within this set there are many genes that confer risk to many different types of traits and many that have a conserved biological function. This is consistent with previous observations that broad functional categories and highly connected genes tend to be enriched for many traits and functional marks^21,28^. As these recurring genes obscure the specific pathways and key genes for a trait, we corrected for this average signal in the downstream analysis to obtain gene-level significance scores that are as trait-specific as possible.

### Downstreamer pinpoints the tissues that contribute to each disease

The next step in the Downstreamer framework was to determine the tissues of interest for each GWAS trait. This was done by linearly associating the gene z-scores for a GWAS to the mean expression in a tissue, while accounting for relationships between genes stemming from LD structure and adjusting for the average GWAS signal (Fig. 1, Methods). The mean tissue-expressions and gene-modules used in Downstreamer were calculated based on a database of gene-expression samples assembled by Recount3^27^. After quality control (QC) and pre-processing (see “Methods” Section), we retained 46,410 samples across 57 primary tissues for which at least 77 (average 814) samples per tissue were available. In each of these tissue-expression matrices, we retained only the genes with sufficient expression in that tissue to ensure that our gene-modules reflect tissue-specific gene regulation and are not biased towards genes showing tissue-specific expression (see “Methods” Section).

We then calculated the average expression per gene for each tissue and associated this to the gene z-scores as a measure of the relevance of that tissue for the trait. For each trait, we used only the tissues that show a Bonferroni-significant enrichment of key gene predictions (Table S4).

### Large-scale tissue-specific gene-expression modules and gene prioritisation using relevant tissues

For tissues with significant enrichment, we determined which gene-modules are associated to the GWAS. The gene-modules for a tissue are given by the eigenvectors of the gene-expression data for that tissue. We linearly associated the gene-level z-scores we obtained from PascalX to the eigenvectors, as described above (see “Methods” Section). Given that eigenvectors are linearly independent, we used the weighted sum of significantly associated eigenvectors (at Bonferroni significance) in a tissue as measure of importance that we call the “key gene score” (Fig. 1, Methods). As phenotypes can manifest in multiple tissues, we meta-analysed the tissue-level z-scores to derive the cross-tissue relevance of a gene, a metric we designate the “meta key gene score” (Table S5). The meta key gene score is essentially a z-score indicating the expected importance of a gene in the tissues enriched for a GWAS. In the analysis below, these meta-analysed key genes scores are used to show the relevance of the prioritised genes.

**Fig. 2:**
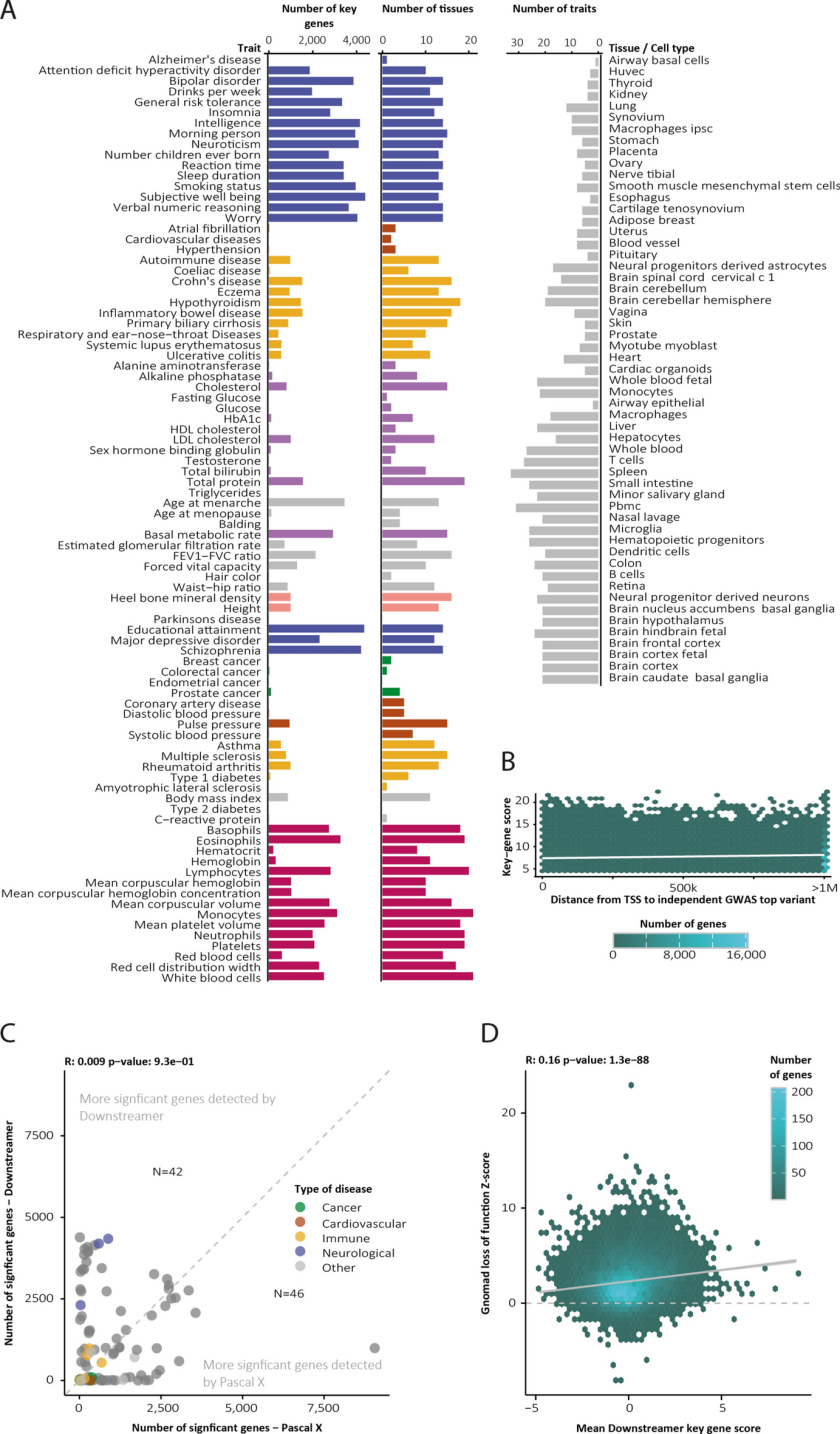
Overview of Downstreamer results across 57 tissues and 88 GWAS traits. (**A**) Summary statistics of Downstreamer output. (**B**) We observed limited association between a gene’s key gene score and the distance between a GWAS top effect and the gene’s transcription start site. (**C**) For some traits, Downstreamer identifies more significant candidate genes. For others, the candidate set is reduced. (**D**) The association between a gene’s average key gene score over the 88 GWAS traits and its intolerance to loss-of-function variants reported in gnomAD.

Hierarchical clustering of the key gene scores revealed that GWAS clustered according to their phenotypic category (as defined in Table S1), with brain-, blood- and immune-related tissues and traits forming distinct clusters (Fig. S1A). We detected the largest number of key genes for brain-related disorders and tissues, possibly due to the distinct gene-expression profiles found in brain tissues (Fig. 2A). Next, we evaluated if genes proximal to GWAS loci were more likely to be key genes than those in *trans* regions. While significant, we observed no strong association between the distance from an independent GWAS signal to the transcription start site (TSS) of the key gene and its key gene score (Fig. 2B, Pearson r = 0.04, *p* < 2.2 × 10^−16^). The slight positive correlation and the fact that the majority (78%) of key gene–trait pairs were located > 500 kb away from an independent GWAS variant suggest that most of the regulation of key genes by common variants is happening in *trans* rather than *cis*.

We subsequently evaluated how many unique genes were identified in this approach compared to using GWAS summary statistics alone (Fig. 2C, Table S6). Here, the identification of significant genes was dependent on the trait, with some traits yielding more results when assaying the GWAS signal alone, while others, notably brain-related traits, showed more candidate genes when assessing key gene scores.

To ascertain if the key genes are likely to have any functional consequence, we associated the key gene scores to conservation metrics from the gnomAD consortium^29^ as a measure of biological relevance. Here we observed moderate correlation between the likelihood that a gene is sensitive to LoF mutations and its average key gene score (Fig. 2D, Pearson r = 0.16, *p* = 1.3 × 10^−88^).

### Human phenotype ontology enrichments for prioritised genes

Downstreamer aims to identify genes that play a key role in complex disease. A strong line of supporting evidence for the relevance of these key genes is if they have a known functional role in rare diseases with a phenotype related to the GWAS traits. We therefore used the enrichment of rare disease genes as a criterion to systematically evaluate genes prioritised by Downstreamer.

**Fig. 3:**
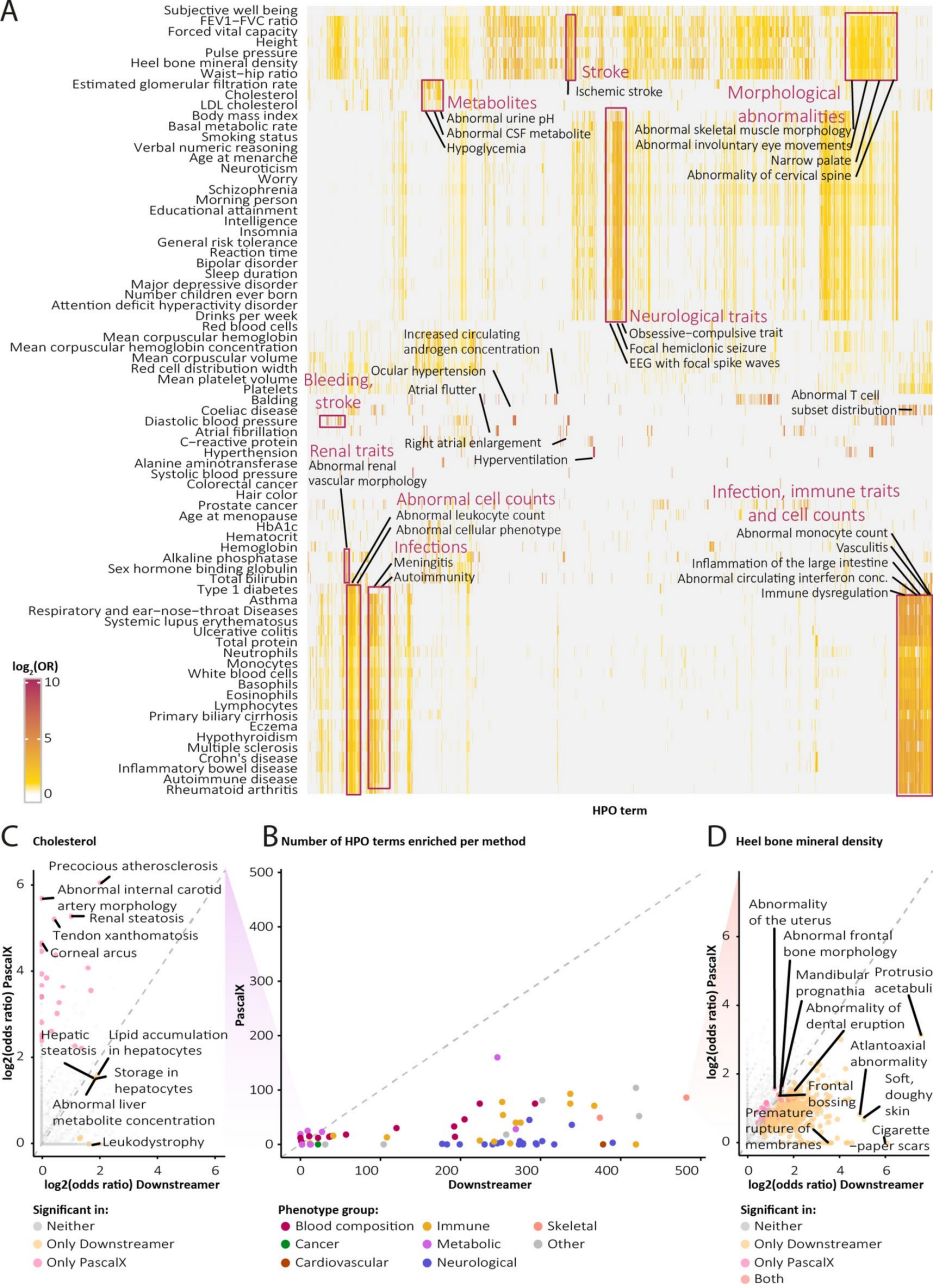
Enrichments of Human Phenotype Ontology (HPO) terms. (**A**) Heatmap of HPO enrichments (rows) per genome-wide association study (GWAS) trait (columns). (**B**) Number of significant HPO enrichments (adjusted *p*-value < 0.05) found for Downstreamer and PascalX. (**C**–**D**) Examples of traits with very few (cholesterol, left) and many (heel bone mineral density, right) HPO enrichments among Downstreamer results. HPO terms labelled.

The Human Phenotype Ontology (HPO) database connects genes to the rare phenotypes that occur when that gene is mutated. Various mutations in the same gene may give rise to different phenotypes, so HPO mappings are not necessarily one-to-one. We calculated the HPO term enrichment for all significant Downstreamer genes, per GWAS trait (see “Methods” Section). This showed that the set of genes identified by Downstreamer is enriched with genes that cause similar phenotypes when mutated (Fig. 3A, Table S7). For example, GWAS autoimmune traits are enriched with HPO terms associated to infection, immune traits and abnormal cell counts. Similarly, neurological and behavioural GWASs, like those for neuroticism, schizophrenia and major depressive disorder, are broadly enriched with brain-related HPO terms. While these general enrichments may reflect tissue-enrichment (Fig. 2), we also observe specific common–rare phenotype pairs. For example, the GWAS for balding is enriched for increased androgen concentration, that for glomerular filtration rate shares genes with urine acidity and the GWAS for atrial fibrillation is connected to right atrial enlargement and atrial flutter (Fig. 3A).

To test whether the gene enrichment of rare disorders is more pronounced in Downstreamer compared to PascalX, which relies only on genes in GWAS loci, we performed the same HPO enrichment for PascalX (Fig. 3B). For nearly all traits, Downstreamer identified more HPO terms. The notable exceptions are a few blood and metabolite traits for which both methods identified a limited number of HPO enrichments. Interestingly, for cholesterol, Downstreamer finds liver-related enrichments, whereas PascalX reflects lipid phenotypes directly (Fig. 3C). On the other hand, for a trait where Downstreamer finds many more enrichments, these additional HPO terms fit the GWAS trait well. For example, heel bone mineral density shares genes with other skeletal phenotypes like protrusio acetabuli (a hip malformation), spondylolisthesis (spine instability) and atlantoaxial dislocation (a neck anomaly) (Fig. 3D).

**Fig. 4:**
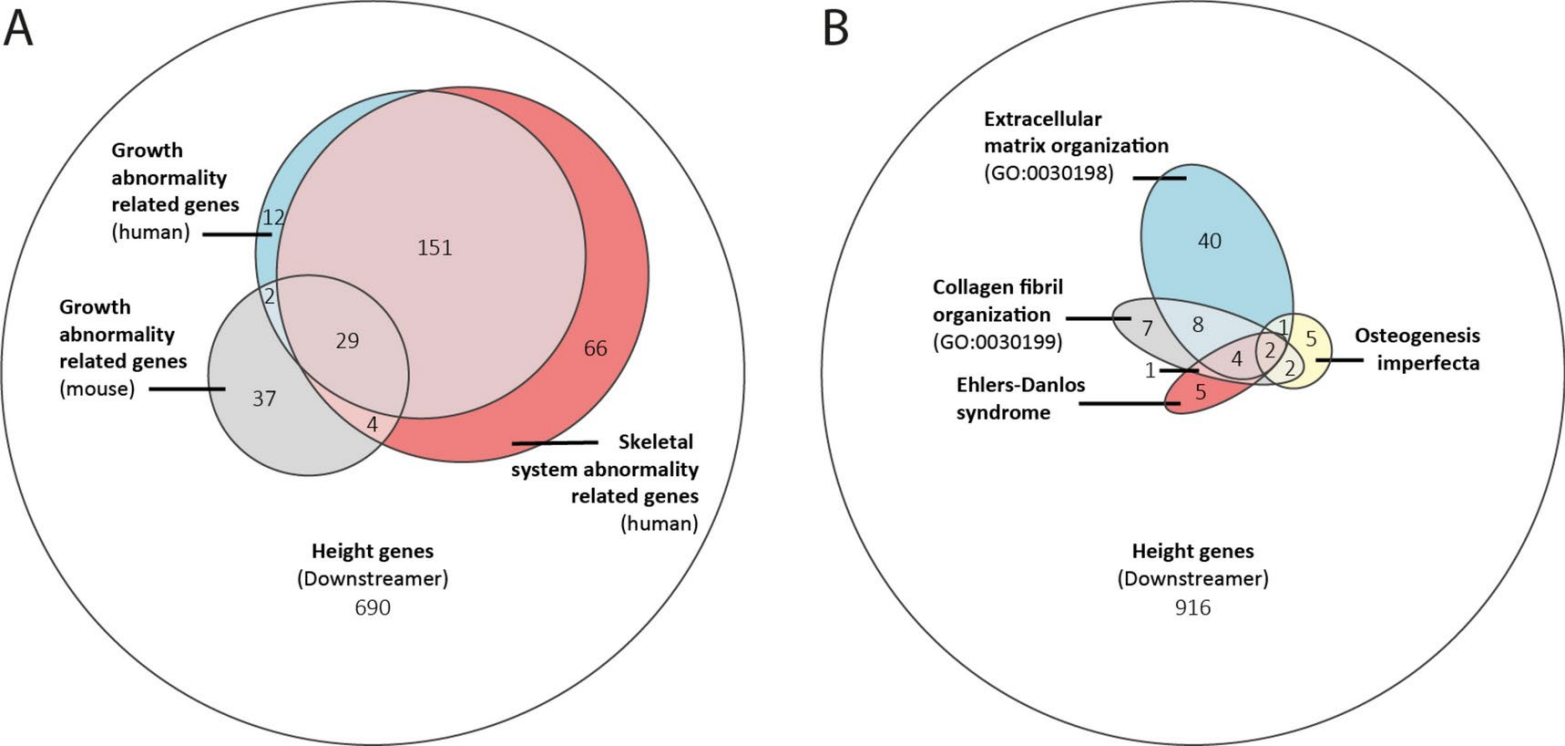
Overlap between Downstreamer-prioritised genes and other gene sets. (**A**) Euler diagram showing the overlap between human and mouse growth-related genes and Downstreamer-prioritised genes for height. (**B**) Euler diagram showing the overlap between Downstreamer-prioritised genes for height; genes associated to two rare diseases with skeletal defects, osteogenesis imperfecta and Ehlers–Danlos syndrome; and genes associated to gene ontology (GO) terms for extracellular matrix organisation and collagen fibril organisation.

In a striking example, the 991 genes prioritised for height show a large overlap with known growth-related genes in human (HPO terms HP: 0001507 and HP: 0000924) and mouse (MGI terms MP: 0002089, MP: 0001730, MP: 0003984, MP: 0010832 and MP: 0005508) (Fig. 4A). We then investigated two diseases with known skeletal defects more closely: Ehlers–Danlos syndrome and osteogenesis imperfecta. Ehlers–Danlos syndrome is a connective tissue disease with 13 subtypes^30^, each with a slightly different set of symptoms and known causal genes. Overall, the syndrome is characterised by skeletal phenotypes, and 12 of the 20 currently known Ehlers–Danlos genes were also prioritised for height by Downstreamer (Fig. 4B). Osteogenesis imperfecta is a group of genetic disorders that lead to structural defects that cause patients’ bones to break easily. Notably, we identify height key genes not currently known to cause Ehlers–Danlos or osteogenesis imperfecta to play a role in these processes, suggesting that these genes could be interesting targets when interpreting variants of unknown significance in patients suspected to have one of these diseases.

## Discussion

We present Downstreamer, a method that integrates cell-type- and tissue-specific gene-modules with GWAS summary statistics to prioritise the genes central in a respective trait’s network. When we applied Downstreamer to 88 GWASs, the genes it prioritised were often not located in the GWAS loci themselves, yet they are good candidates based on pathway annotation and their involvement in severe (Mendelian) forms of these diseases. These key genes have different characteristics than the positional candidate genes: they are enriched for being evolutionarily constrained, indicating that they more often have crucial biological functions. These findings suggest that the small effects of GWAS-associated variants ultimately converge on key disease genes.

We also observed that the gene-prioritisation scores of related traits are often correlated (Fig. S1A). The genes with a high score for one trait have higher-than-expected by chance scores for similar traits. To some extent this is reasonable given the known shared genetic signature of, e.g. autoimmune disorders. However, if we had used co-expression calculated in a multi-tissue dataset, our predictions might have only reflected expression in a relevant tissue. To overcome this, we used tissue-specific gene-modules where the co-expression is not confounded by tissue-specific expression. This makes it likely that the shared key genes we identified reflect shared aetiology of these diseases. However, we do note that cell-type-specific expression within a tissue might still drive some of the enrichments we observe.

We recently applied a pre-release version of Downstreamer to several neurodegenerative diseases while using a comprehensive brain-specific gene co-regulation network of the MetaBrain project. This revealed that the signal of underrepresented cell types and tissues can be overshadowed by the more abundant tissues in the expression data^31^. This might be especially relevant for diseases where uncommon or rare cell types, e.g. regulatory CD4 + T cells^32^, are instrumental to disease pathophysiology. As we have shown using 57 tissue- and cell-type-specific gene-modules, we expect that key gene prioritisation will benefit from more cell-type-specific co-expression networks, should enough samples be available for the relevant tissue and cell type to accurately calculate these networks. We believe that single-cell RNA-seq datasets will soon enable generation of such co-expression datasets, provided that sufficient numbers of cells are studied to accurately quantify co-expression, particularly for genes with low expression.

One assumption we make when calculating the gene p-values is that causal variants map within 25 kb of the associated gene, which might not be the case for all genes. However, recent work has suggested that, except for integrating epigenetic and HiC contact data, the next best predictor of causality is the gene closest to the top GWAS signal, and this approach outperforms eQTL-based approaches^33^. We therefore decided not to integrate any prior eQTL information when calculating gene *p*-values, as this would often lead to incorrect prioritisations. In addition, the genes affected by GWAS variants are also likely to be tissue-specific, further complicating the prioritisations, and we would need extensive prior information to select the correct eQTLs or epigenetic information. This therefore represents an area where major improvements in the future would enable accurate and systematic predictions about which genes are regulated by GWAS variants in *cis.*

Downstreamer uses LD information from an external reference panel in the PascalX step to get the initial gene *p*-values. As the GWAS studies we used in this study are predominantly performed in European populations, we used the European samples of the 1000 Genomes data as reference. For GWAS studies on other populations, we recommend using matching LD information.

Our findings are in line with the infinitesimal model^34^, which postulates that a quantitative trait or complex genetic disease can result from an infinite number of variants, each exerting an infinitely small effect size. The omnigenic model^21^ is thus an extension of the infinitesimal model, as it predicts that all genes expressed in the relevant tissue or cell type will have a non-zero effect on disease outcome. The omnigenic model also postulates the existence of core genes that are pivotal in the development of a disease or trait. These core genes are expected to be enriched for genes involved in rare Mendelian diseases. The fact that key genes tend to be highly expressed in the relevant tissues for a trait, together with the enrichments of rare disease genes among the key genes, fits the regulatory pattern hypothesised in the omnigenic model. Hence, at least some of the key genes we predict using Downstreamer could be the core genes described in the omnigenic model.

The enrichment of Downstreamer key genes among known Mendelian disease genes that we observed has important implications for rare disease diagnostics. At present, a genetic cause is identified for only 30% of patients suspected to have a rare disease, on average^35^. One reason for this low diagnostic yield is that when a rare variant is found in a gene with an unknown function, it is difficult to determine whether this variant is causative for a patient’s phenotype. We expect that approaches like ours could eventually be used to leverage the key genes of common diseases and traits to prioritise candidate rare disease genes, similar to what we did previously using GADO^36^.

In summary, we have presented Downstreamer, a method that integrates multi-tissue gene regulatory networks with GWAS summary statistics to prioritise key genes central in the gene network. We found that these key genes are enriched for Mendelian variants that cause related phenotypes, highlighting that GWAS signals partially converge on Mendelian disease genes. While gaps remain in our understanding of the *trans* regulatory architecture of GWAS traits and diseases, assessing the genes most central in their respective regulatory network presents a promising way forward for interpreting both complex and rare disease genetics.

## Methods

### Datasets and pre-processing

#### GWAS summary statistics

We downloaded the publicly available summary statistics from either the GWAS catalogue^37^ or the supplementary data files. A full list of the summary statistics used is available in Table S1. As Downstreamer requires the rs identifiers (RsId) of the variants as well as the p-values, we extracted these from the summary statistic files and removed any duplicate variants and variants without a RsId. Where needed, the summary statistics were lifted to build 37 and the RsIds matched on position and allele to 1000 Genomes phase 3 EUR for all variants with a minor allele frequency (MAF) > 0.05^38^. In addition, we integrated summary statistics from a curated database of traits^18^, expanding the total scope to 88 traits. No further processing other then MAF filters was performed on this set of summary statistics.

#### Pathway databases

We used the following pathway and gene-set databases: Reactome^39^, KEGG^40^, GO^41^ (downloaded July 18, 2020), HPO^42^ (filtered version as in^43^) and MGI (downloaded October 20, 2020)^44^.

#### Gene-expression data

The Recount3 data we use to create our multi-tissue network and tissue-specific networks was collected by Wilks et al*.*^27^ This datasets contains 316,443 human RNA-seq samples that have been uniformly processed and quantified. We performed additional QC and predicted if the samples are primary tissue, cell line or cancerous. This allowed us to select 58,725 samples expected to be primary tissues (Note S1).

We then predicted the tissues of origin for samples lacking annotations. Next, we performed QC per tissue, thereby reducing the total number of available samples to 46,410 for 57 different tissues and cell types (Note S2). For each tissue, we selected genes expressed in at least 50% of the samples and performed variance stabilizing transformation (VST)^45^ normalisation. We then regressed out technical confounders as before (Note S1) to obtain final tissue matrices. For the full Recount3 expression data, we use the same 46,410 samples that passed the per-tissue QC but using quantile normalisation instead of VST.

For the full Recount3 expression matrix and the tissue-specific matrices, we used singular value decomposition on the per-gene-scaled expression data to obtain the eigenvectors with the gene loadings. For the full matrix, we selected the eigenvectors that jointly explain 85% of the variance (n = 847). For the tissues, we confined ourselves to eigenvectors that explain 80% of the variance.

### Overview of Downstreamer framework

The following describes the key steps in the Downstreamer framework. In short, variant-level summary statistics are converted to gene-level z-scores using PascalX (Step 1). We then use these to determine the average likelihood that a gene is associated to any GWAS trait (Step 2). Next, we calculate gene–gene relationships stemming from LD (Step 3). We then determine relevant tissues for a GWAS trait and associate gene-modules, accounting for gene–gene relationships and the average likelihood of GWAS association from Step 2 (Step 4). We use the enriched gene-modules to determine key gene scores that describe genes with relevance over several modules within and across relevant tissues (Step 5). Finally, we overlap key gene scores with phenotype annotations to identify a set of core genes with likely regulatory effect and a known phenotypic association (Step 6). These we call key genes.

#### Step 1: conversion of variant-level summary statistics to gene z-scores

Variant-level p-values for each of the summary statistics were converted to gene-level p-values using PascalX^26^. In brief, PascalX aggregates variant-level p-values in a window around a gene (we used 25kb up- and downstream of the gene body) while accounting for the LD structure in the locus. We used 1000 Genomes EUR phase 3 non-Finnish samples as the LD reference panel. We used ENSEMBL version 94 to define the gene boundaries, calculating gene p-values for all protein-coding genes. These *p*-values were then converted to z-scores.

#### Step 2: determining shared signal among GWAS traits

We and others have observed that GWAS signals are not randomly distributed among genes. Certain genes tend to be more likely to harbour GWAS variants, in particular around variants regulating transcription. However, such signals give rise to downstream enrichment that is not specifically related to the biology of the GWAS trait, thus complicating interpretation. To correct for this shared signal, we took the average PascalX gene z-score over all the traits we test as a measure of how likely a gene is to be associated to a GWAS trait. To not skew this average towards the mean highly similar traits that are over-represented in our dataset (e.g. immune cell counts in blood), we calculated the average gene z-score in two stages. First, we calculated the average gene z-score for a gene per class of GWAS trait (immune, cancer, skeletal, blood composition, neuro, cardiovascular, metabolic, other). Next, we calculated the average over these classes, thereby ensuring they were weighted equally. This vector containing the “mean of means” was used for subsequent analysis.

#### Step 3: calculation of relationships between gene z-scores due to LD

Given the LD structure between nearby variants, a similar correlation structure permeates the gene *p*-values we used to run enrichments. To quantify this correlation structure, we first simulated 10,000 random phenotypes by drawing phenotypes from a normal distribution and associating them to the genotypes of the 1000 Genomes EUR phase 3 non-Finnish samples. We then calculated the GWAS gene p-values and converted them to z-scores for each of the 10,000 simulated GWAS signals, as described above. Next, we calculated the Pearson correlations between the GWAS gene z-scores per chromosome arm separately, setting all correlations across chromosome arms to zero because we assume there is no LD across chromosome arms. We refer to this correlation matrix as Ω. As the simulated GWAS signals are random and independent of each other, any correlation between these GWAS gene z-scores reflects the underlying LD patterns and the chromosomal organisation of genes.

#### Step 4: linear association of GWAS gene z-scores with expression eigenvectors and pathways

Next, we associated the gene z-score profiles and phenotype of interest. In this regression, we treated the gene z-score profile as the response variable. First, the gene z-scores for a GWAS were transformed into a normal distribution using an inverse normal transformation. We refer to this variable as y. This normalised vector is then associated to the phenotype (dubbed x) in an approximate generalised least square (GLS)-based framework. The phenotype x can either be pathway membership (coded as a dummy variable) or any normally distributed continuous variable. For the key gene prioritisations, we used the eigenvectors derived from the tissue-specific expression data, described above as x. We note that the implementation of this strategy shares similarities with MAGMA^10^ but is not identical. As covariates C, we used the shared baseline signal from Step 2 and gene length.

As the gene–gene correlation matrix Ω that we derive in Step 3 is not guaranteed to be positive-definite, rather than deriving the inverse of this matrix to run GLS, we ran an approximate GLS through the eigen decomposition of Ω. First, we intersected all available genes between y, x, Ω and any covariates C. We then ran an eigen decomposition on Ω.

$$\Omega = \lambda V$$

We then selected all eigenvalues and eigenvectors explaining 90% of the variance in Ω. We call the reduced vector and matrix containing the eigenvalues and eigenvectors λ′ and V′, respectively. This step removes noise that would otherwise be introduced by incorporating eigenvectors with very small eigenvalues describing noise.

We note that the following equations are equivalent to GLS in the case where all eigenvectors are used to apply the transformation^46^. However, this can only be done if Ω is positive-definite. As Ω is not positive-definite in practice, we did not use all eigenvectors, so we refer to this approach as “approximate GLS”. In practice, the eigenvectors we leave out will describe noise and including them has a detrimental effect on the results (Fig. S4). We simulated random phenotypes containing a strong correlation structure, and these simulations showed this approach can account for the correlation structure while maintaining well-calibrated p-values under the null (Fig. S4).

We then multiplied y, x and C by the transpose of V′ to remove the correlation structure attributable to LD.

$$\begin{aligned} y^{\prime } = & V^{\prime T} y \\ x^{\prime } = & V^{\prime T} x \\ C^{\prime } = & V^{\prime T} C \\ \end{aligned}$$

Here, y′, x′ and C′ represent the rotated gene z-scores, phenotype and covariates, respectively. We then merged x′ and C′ into the design matrix X′, adding an intercept term. The regression betas are then derived as follows:

$$\beta = { }(y^{\prime T} \Lambda^{\prime - 1} X^{\prime } ){ }\left( {X^{\prime T} \Lambda^{\prime - 1} X^{\prime } } \right)^{ - 1}$$

where β represents the column vector of effect sizes for the design matrix X′ and Λ′^−1^ is a diagonal matrix containing the inverse of the eigenvalues in λ′. The standard errors for the regression estimates are estimated as follows:

$$e = y^{\prime } - X^{\prime } \beta^{T}$$

where e represents the vector of regression residuals

$$rss_{w} = \frac{{\left( {e^{T} \Lambda^{\prime - 1} e} \right)}}{df}$$

where the numerator is the residual sum of squares and df is the degrees of freedom.

$$\beta_{se} = \sqrt {{ }\left( {X^{\prime T} \Lambda^{\prime - 1} X^{\prime } } \right)^{ - 1} {* }rss_{w} }$$

where β_se_ is the vector of standard errors for the elements in β. *P*-values for the effect of x′ are then estimated by deriving the T-statistic (β/β_se_) and the inverse cumulative of the T-distribution with degrees of freedom df. These estimates of β and β_se_ are independent of the LD structure given Ω is an appropriate proxy of the true LD structure.

#### Step 5: determining key gene scores

To determine key gene scores for a GWAS tissue pair, we took the g × m matrix of significantly associated eigenvectors V_tissue_ for each tissue and the vector β_eigen_ with their corresponding effect sizes from Step 4 and calculated a weighted sum. Here, g is the number of genes and m is the number of significantly associated eigenvectors.

$$V_{sum} = { }\mathop \sum \limits_{i = 0}^{m} V_{{tissue{ }i}} { }\beta_{{eigen{ }i}}$$

where V_sum_ is a vector of length g containing the weighted sum over the eigenvectors for each gene. We note that the arbitrary directions assigned to eigenvectors are accounted for through the sign of their corresponding β_eigen_.

We then determined the significance of the elements in V_sum._ First, we created 10,000 V_sum_ values per gene as null distribution using the same significant eigenvectors but now using random values for β_eigen_ samples from a normal distribution with mean 0 and sd 1. Then, for each gene, we used the probability density function of the Johnson SU-distribution using the mean, sd, kurtosis and skewness of the 10,000 V_sum_ of the permutations of this gene to obtain a *p*-value.

We used this p-value to derive a vector of z-scores (Z_tissue_) for each gene, which we dub the key gene score for that tissue. We calculated Z_tissue_ for each GWAS and all k significantly enriched tissues to that GWAS to derive a g × k matrix Z_all_ of tissue-specific z-scores.

As an overall measure of association of a gene to a GWAS, we performed a meta-analysis using Stouffer’s method, ascribing equal weights to every tissue. The k individual per tissue vectors are used to derive a vector Z_meta_ describing the overall association of genes to the GWAS.

$$Z_{meta} = \frac{{\mathop \sum \nolimits_{i = 0}^{k} (Z_{{all{ }i}} )}}{\sqrt k }$$

where Z_meta_ is a vector of length g giving the meta z-score for each gene and k is the number of tissues significantly enriched for the GWAS trait, excluding the multi-tissue dataset.

### Downstream analysis

#### Simulation of null dataset

Using the gene–gene correlation matrix Ω derived in Step 3, we selected genes showing strong average correlation to other genes. From this set, 300 genes were randomly selected. This correlation matrix of 300 × 300 genes was then transformed to ensure it is positive-definite using the function ‘nearPD’ from R package Matrix^47^, and we refer to this matrix as Ω′. Using Ω′, we simulated 1000 random traits with the correlation structure described by Ω′ using a multivariate normal distribution implemented in mvnnorm from R package MASS^48^. The same function was used to simulate a random GWAS trait with no association to the 1000 pathways other than that introduced through Ω′. This dataset was then used as input for an ordinary least squares (OLS) model where the correlation is not taken into account, for the Downstreamer model and for a GLS, as well as being used as a benchmark for calibration of p-values under the null when data is highly correlated (Fig. S4).

#### Clumping of GWAS summary statistics and determination of closest genes

Variant *p*-values from the individual GWASs were retrieved and clumped using PLINK 1.9^49^. We used a window of ± 500kb, an r2 threshold of 0.1 and a *p*-value threshold of 5 × 10^−8^ to make clumps. The 1000 Genomes non-Finnish samples with variants at a MAF > 0.05 were taken as the LD reference panel. These clumps were used as input to determine the closest genes and TSSs. Closest genes were determined by filtering the ENSEMBL 94 annotations for protein-coding genes, after which the closest genes were determined using the function ‘nearest’ from the GenomicRanges package in R^50^. The same was done for TSSs. The TSS positions were defined as the outermost position of the gene, taking its orientation into account. No alternative transcript usage was considered.

#### Enrichment of key genes

Enrichments of key genes among HPO/MGI/GO terms and KEGG gene sets was done by Fisher’s exact test, taking all key genes at Bonferroni or false discovery rate significance and comparing their overlap with all other genes. Area under the curve values were calculated by dividing the Mann–Whitney U statistic of the key gene z-scores and gene-set membership by the product of sample sizes. The gene pathway/term definitions we used were those provided by the respective databases. This is implemented in Downstreamer using—T PRIO_GENE_ENRICH.

To calculate the enrichment of significant Downstreamer genes in HPO terms, we downloaded the HPO phenotype–gene links (V1268, OMIM and ORPHA), selecting only the genes that were part of the Downstreamer analysis. We then excluded HPO terms that had fewer than 10 genes connected to them. For each remaining term, we performed a Fisher’s exact test of key genes (i.e. with a Bonferroni-significant key gene score) versus phenotype-linked genes (i.e. annotated to this HPO term), resulting in an OR and a p-value. We corrected the p-values for multiple testing using the Bonferroni correction (p-value/number of HPO terms).

#### Enrichment of average gene z-scores and association with LD and gene density

Enrichments of the top 500 average gene z-scores were done by first correcting the average gene z-score vector (see Step 2 for details on calculating this) for the extent of the LD around a gene as well as the gene density. To quantify the extent of the LD block, we took the average of the LD scores of all SNPs in a 25kb window around the gene. Pre-computed European LD scores were downloaded from https://github.com/bulik/ldsc. Gene density was calculated by counting the number of genes in a 250kb window around the start and end of the gene. Both these variables were then fit in a linear model with the average gene z-score as the outcome. The residuals were taken and ranked to arrive at the top 500 genes. We then carried out overrepresentation analysis using https://toppgene.cchmc.org/enrichment.jsp with the background set of all protein-coding genes for which we have a gene z-score available. Results are shown in Table S3.

#### Association with LoF and MiS intolerance

MiS and LoF intolerance z-scores were downloaded from the gnomAD consortium (https://gnomad.broadinstitute.org/downloads > pLoF Metrics by Gene TSV v2.1.1). As an overall measure of the “keyness” of a gene, for each gene we calculated the maximum key gene score observed over the 88 traits. We then associated this to the MiS and LoF z-scores from the gnomAD consortium by Pearson correlation.

#### Comparison between Downstreamer results and PascalX

Downstreamer aims to find key genes that play a role in complex disease. The strongest evidence that the Downstreamer key gene score identifies important genes is if high-scoring genes also play a role in rare phenotypes for a related phenotype. We therefore used the enrichment of rare disease genes as a criterion to systematically evaluate Downstreamer in comparison to PascalX.

The HPO database connects genes to rare phenotypes that occur when that gene is mutated. As various mutations in the same gene may give rise to different phenotypes, HPO mappings are not necessarily one-to-one. Moreover, the HPO phenotypes are structured hierarchically, e.g. ‘Abnormal mitral valve morphology’ is a parent term of ‘Mitral valve prolapse’ and they share many of the same genes. To calculate the enrichment of prioritised genes in HPO terms, we downloaded the HPO phenotype–gene links (V1268, OMIM and ORPHA). To calculate the enrichment in Downstreamer results, we first selected only genes that were part of the Downstreamer analysis from the HPO database. We then excluded HPO terms that had fewer than 10 genes connected to them. For each remaining term, we performed a t-test of key genes (i.e. with a Bonferroni-significant key gene score) versus phenotype-linked genes (i.e. annotated to this HPO term), resulting in an OR and a p-value. We corrected the p-values for multiple testing using Bonferroni (p-value/number of HPO terms). We then performed the same enrichment analyses for the genes deemed significant by PascalX.

## Supplementary Information

Supplementary Information 1.

Supplementary Information 2.

Supplementary Information 3.

Supplementary Information 4.

Supplementary Information 5.

Supplementary Information 6.

Supplementary Information 7.

Supplementary Information 8.

Supplementary Information 9.

## Data Availability

Software and scripts are available for download at: https://github.com/molgenis/systemsgenetics/tree/master/Downstreamer. A manual for Downstreamer is available at: https://github.com/molgenis/systemsgenetics/wiki/Downstreamer. All RNA-seq data used in the main analysis are publicly via the Recount3 website at: https://rna.recount.bio/
