# Supplementary material for: Identification of rare disease genes as drivers of common diseases through tissue-specific gene regulatory networks
Source: Sci Rep. 2024 Dec 4;14:30206. doi: 10.1038/s41598-024-80670-1 (PMC11618476; doi:10.1038/s41598-024-80670-1)
Supplement: Supplementary file 1 — Supplementary Information 1. [file 41598_2024_80670_MOESM1_ESM.pdf]

Supplementary data for: Identification of rare disease genes as drivers of common diseases through tissue-specific gene regulatory networks

Note S1. Quality control and sample selection of Recount3 data

Note S2. Tissue prediction and per tissue quality control of Recount3 data

Fig. S1. Association between PascalX gene z-score profiles of different traits

Fig. S2. Association between average PascalX gene z-score and LD and gene density

Fig. S3. Enrichment of missense and loss of function intolerance in the average PascalX gene z-score

Fig. S4. Results on null data with correlation structure and evaluation of optimal threshold for eigenvector selection

Table S1. List of the 88 complex traits and diseases to which we applied Downstreamer

Table S2. List of included tissues

Table S3. Enrichment of top 500 genes from average GWAS signal

Table S4. Significant tissues per trait

Table S5. Significant key genes (Downstreamer) per trait

Table S6. Significant GWAS genes (PascalX) per trait

Table S7. HPO enrichments of key (Downstreamer) genes per trait

Note S1. ***Quality control and sample selection of Recount 3 data***

Phase 1: We first performed a basic quality control (QC) of samples using the following steps.

- Remove samples annotated as single cell (n=74,412).
- Remove 4SU-labelled samples (n=1,589).
  - Mention of '4su' or 'thiouridine' in one of these columns: "sra.library_construction_protocol"; "sra.study_abstract"; "sra.experiment_title"; "sra.design_description"; "sra.sample_description"; "sra.library_construction_protocol"; "sra.sample_attributes"; "sra.sample_title"
- Remove samples with only NaN expression values (n=239).
- Remove samples with missing metadata (n=1,711).
- Exclude samples based on the following QC metrics (n=96,597):
  - sra.sample_spots — <1e6 or >2e8
  - for TCGA samples, recount_qc.bc_frag.count — <1e6 or >2e8
  - recount_qc.star.uniquely_mapped_reads_% — <60%
  - recount_qc.aligned_reads%.chrm — >20%
  - recount_qc.aligned_reads%.chrx — >6%
  - recount_qc.aligned_reads%.chry — >0.5%
  - recount_seq_qc.%n — >2%
  - recount_seq_qc.%a — <20% or >35%
  - recount_seq_qc.%c — <20% or >35%
  - recount_seq_qc.%g — <20% or >35%
  - recount_seq_qc.%t — <20% or >35%
  - recount_qc.star.%_of_reads_mapped_to_too_many_loci — >0.5%
  - recount_qc.junction_count — >500,000
  - recount_qc.star.deletion_average_length — >3
  - recount_qc.star.number_of_splices:_total — <150,000
  - recount_qc.intron_sum_% — >20
  - recount_qc.bc_auc.unique_% — <125
- Exclude all data from study SRP025982 (mixed tissues and spiked data for benchmarks).

Phase 2: We only retained genes that were expressed in at least 50% of the samples.

Phase 3: We performed another sample QC using only the maintained genes.

- Exclude samples with 0 expression >50% of the genes.
- Remove duplicate samples.
- Exclude samples with 0 variance.
- Use singular value decomposition (SVD) on quantile-normalised expression to remove outliers on the first component.

Phase 4: We corrected the remaining samples for covariates using the following steps.

- Correct the expression data for the following technical covariates:
  - recount_seq_qc.avg_len
  - sra.sample_spots
  - recount_qc.bc_frag.count

    - recount_qc.star.uniquely_mapped_reads_%
    - sra.library_layout
    - recount_qc.aligned_reads%.chrm
    - recount_qc.aligned_reads%.chrx
    - recount_qc.aligned_reads%.chry
    - recount_seq_qc.%a
    - recount_seq_qc.%c
    - recount_seq_qc.%g
    - recount_seq_qc.%t
    - recount_qc.bc_auc.unique_%
    - recount_qc.intron_sum_%
    - recount_qc.star.%_of_reads_mapped_to_too_many_loci
    - recount_qc.junction_count
    - recount_qc.star.deletion_average_length
- 675 SRA samples were excluded due to missing covariate data. The total number of samples included was 142,849.

Phase 5: We predicted cell lines and cancer samples. The predictions were based on the sample principal components and trained using the annotations known for a subset of the samples. For the prediction of primary tissues vs cell lines, we used logistic regression using the principal components.

For the prediction of cancer samples, we used the method developed by Fehrmann *et al.* [51]. This first determines the auto-correlation per component, which is higher for components that reflect copy number alterations. The sample loadings are then used to create a score per sample that indicates the number of copy number alterations in the samples. We then used this score in a second logistic regression model that discriminated between primary tissues and cancer samples.

Neither of these models yielded perfect separation between the three classes of samples. While this is in part driven by erroneous annotations in the public repositories, it did allow us to select samples that are likely to be primary tissues or cell types.

51. Fehrmann, R. S. N. *et al.* Gene expression analysis identifies global gene dosage sensitivity in cancer. *Nat. Genet.* **47**, 115–125 (2015).

Note S2. ***Tissue prediction and per-tissue quality control of Recount3 data***

To predict tissues for the samples that are predicted not to be cell lines or cancerous, we started anew with transcripts per million values. We selected the genes expressed in at least 50% of the samples, performed log2 and quantile normalisation and corrected for the same covariates as before. We then performed a new principal component analysis and used the components in a multinominal logistic regression model trained on the known sample annotations.

One major confounder with tissue type is the associated study. Typically, samples from the same study are sequenced using the same type of sequencer and read length, and most studies investigate a single tissue. But there are many differences among the different studies. We can correct for these to some extent by including technical differences as confounders, but we found that this adversely affected our prediction accuracy. We therefore devised the following strategy to

create a representative training set. Ideally, we would only use a single sample per tissue from each study to train the prediction model. In practice, for some tissues, this would result in a rather limited number of usable samples. To overcome this, we increased the number of samples per tissue per study to ensure at least 50 training samples per tissue. Based on early tests, we noticed that we could not reliably discriminate between adipose and breast samples. These samples were therefore combined in a single adipose-breast network that we refer to as a 'breast' network in this manuscript for clarity.

We then used the R package *glmnet* [52] to do lasso regression with cross validation to select an optimal lambda. This model was then applied to all samples, and we assigned each sample the tissue with the highest posterior probability. Samples for which the highest posterior probability was less than 0.5 were excluded.

As a final quality control, we performed a principal component analysis per tissue and excluded outliers. This resulted in 46,410 samples. Per tissue, we eventually used VST [45] for the normalisation and corrected the data for the covariates. A SVD was used to extract the eigenvectors with gene loadings that are used by Downstreamer for the gene prioritisation.

For the Recount3 multi-tissue network, we used quantile normalisation and covariate correction for the 46,410 samples for which we have a predicted tissue assignment. Here we used SVD to obtain the eigenvectors.

52. Friedman, J. H., Hastie, T. & Tibshirani, R. Regularization Paths for Generalized Linear Models via Coordinate Descent. *J. Stat. Softw.* **33**, 1–22 (2010).

Fig. S1. Association between PascalX gene z-score profiles of different traits

Provided separately.

A) Pearson correlations between gene z-scores reveal that most pairwise correlations between traits are positive. B) Mean correlation in gene z-scores with all other GWASs (y-axis) versus the number of samples in the GWAS (x-axis). C) Mean correlation in gene z-scores with all other GWASs (y-axis) versus the number of independent genome-wide significant hits for the respective GWAS determined by clumping ±500kb window and an r2 of 0.1.

Fig. S2. Association between average PascalX gene z-score and LD and gene density

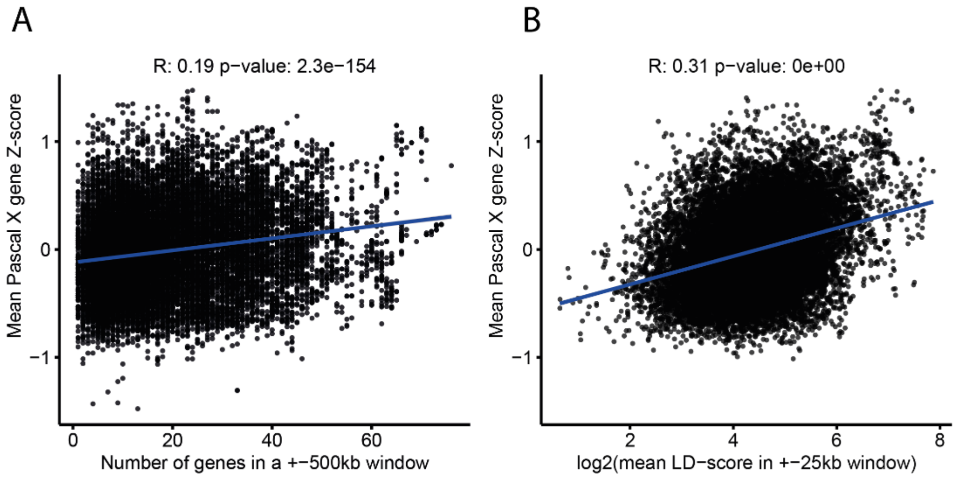

A) Association between average gene z-score (y-axis) and the number of genes within a ±500kb window (x-axis). B) As in (A), but x-axis indicates the log2 of the average LD score of SNPs located ±25kb around the start and end of a gene. The adjusted r2 of the model associating the average gene z-score and these two parameters as the independent variables is 0.147. p-value < 1e-16.

Fig. S3. Enrichment of missense and LoF intolerance in the average PascalX gene z-score

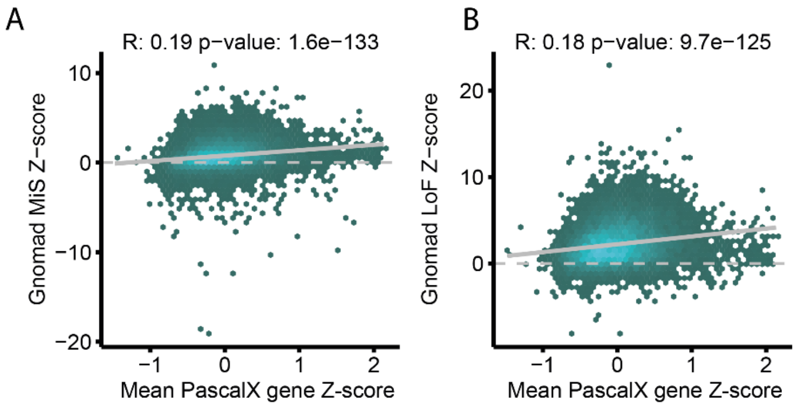

A) Association between average gene z-score (x-axis) and the missense intolerance z-scores from the gnomAD consortium (y-axis). B) As in (A), but the y-axis indicates the z-score for LoF intolerance.

Fig. S4. Results on null data with correlation structure and evaluation of optimal threshold for eigenvector selection

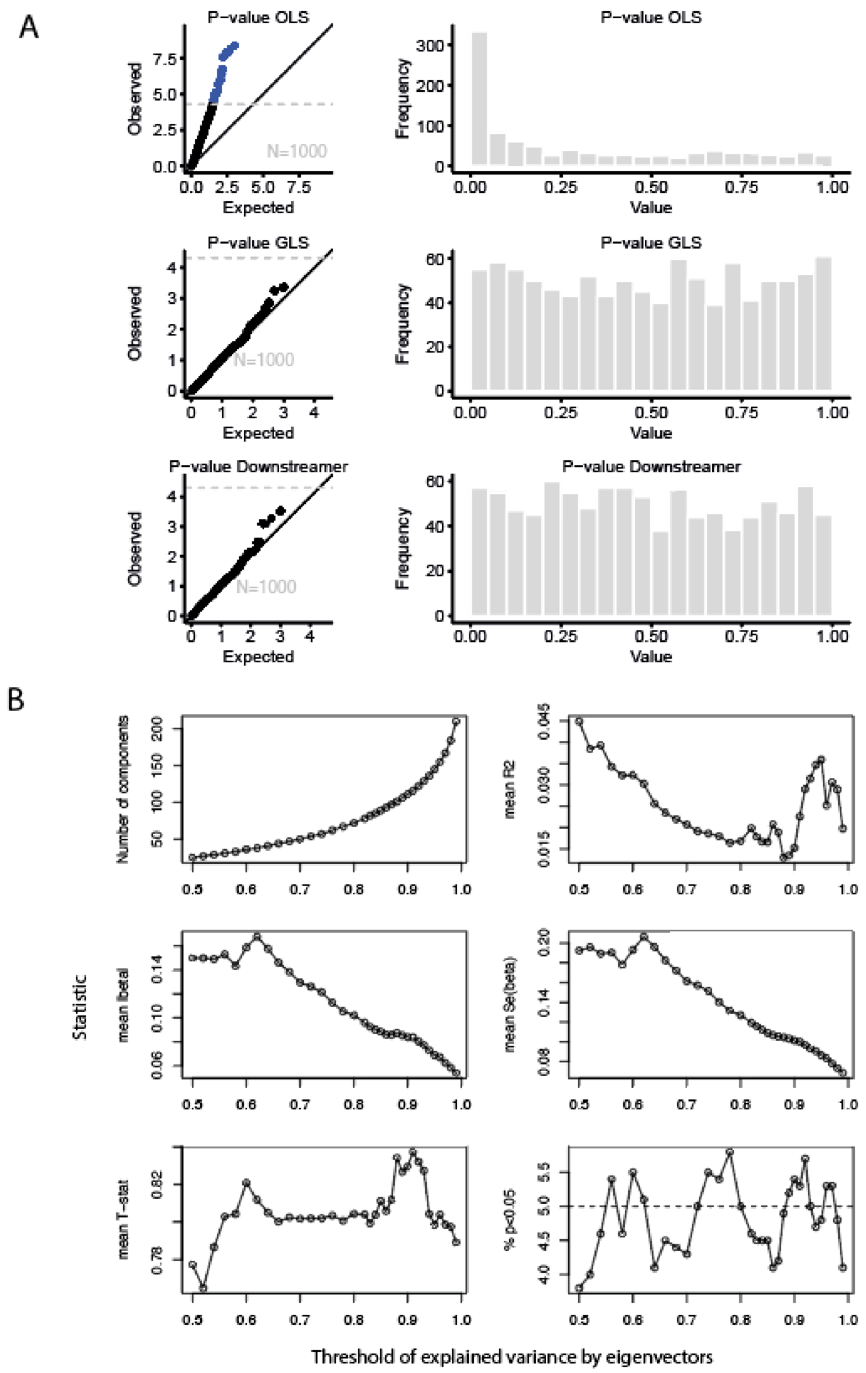

A) Results on simulated data with representative correlation structure for 1000 randomly generated phenotypes. From top to bottom: OLS model, GLS model and Downstreamer model. Both Downstreamer and GLS produce well-calibrated p-values under the null model. B) Evaluation of the optimal number of eigenvectors to use in the approximate GLS model. Evaluated here for the same simulated data as A, mean statistics for the 1000 pathways are shown on the y-axes, except for the first plot which shows the number of eigenvectors, representing the degrees of freedom +1. X-axis shows the percentage of variance explained by the eigenvectors. Y-axes show different statistics on the output. A threshold of 0.9 was chosen as it yielded optimal power, a mean model r2 close to zero, low standard errors and higher T statistics. We note that the simulated data had positive-definite correlation structure, but on real data, inclusion thresholds above 0.9 tend to give rise to inflation as their eigenvalues are approaching the precision limit or are negative.

Table S1. ***The 88 complex traits and diseases to which we applied Downstreamer***

Table S2. ***List of tissues included***

Table S3. ***Enrichment of top 500 genes from average GWAS signal***

Table S4. ***Significant tissues per trait***

Table S5. ***Significant key genes (Downstreamer) per trait***

Table S6. ***Significant GWAS genes (PascalX) per trait***

Table S7. ***HPO enrichments of key (Downstreamer) genes per trait***
