# Supplementary figures and images for: Identification of rare disease genes as drivers of common diseases through tissue-specific gene regulatory networks
Source: Sci Rep. 2024 Dec 4;14:30206. doi: 10.1038/s41598-024-80670-1 (PMC11618476; doi:10.1038/s41598-024-80670-1)

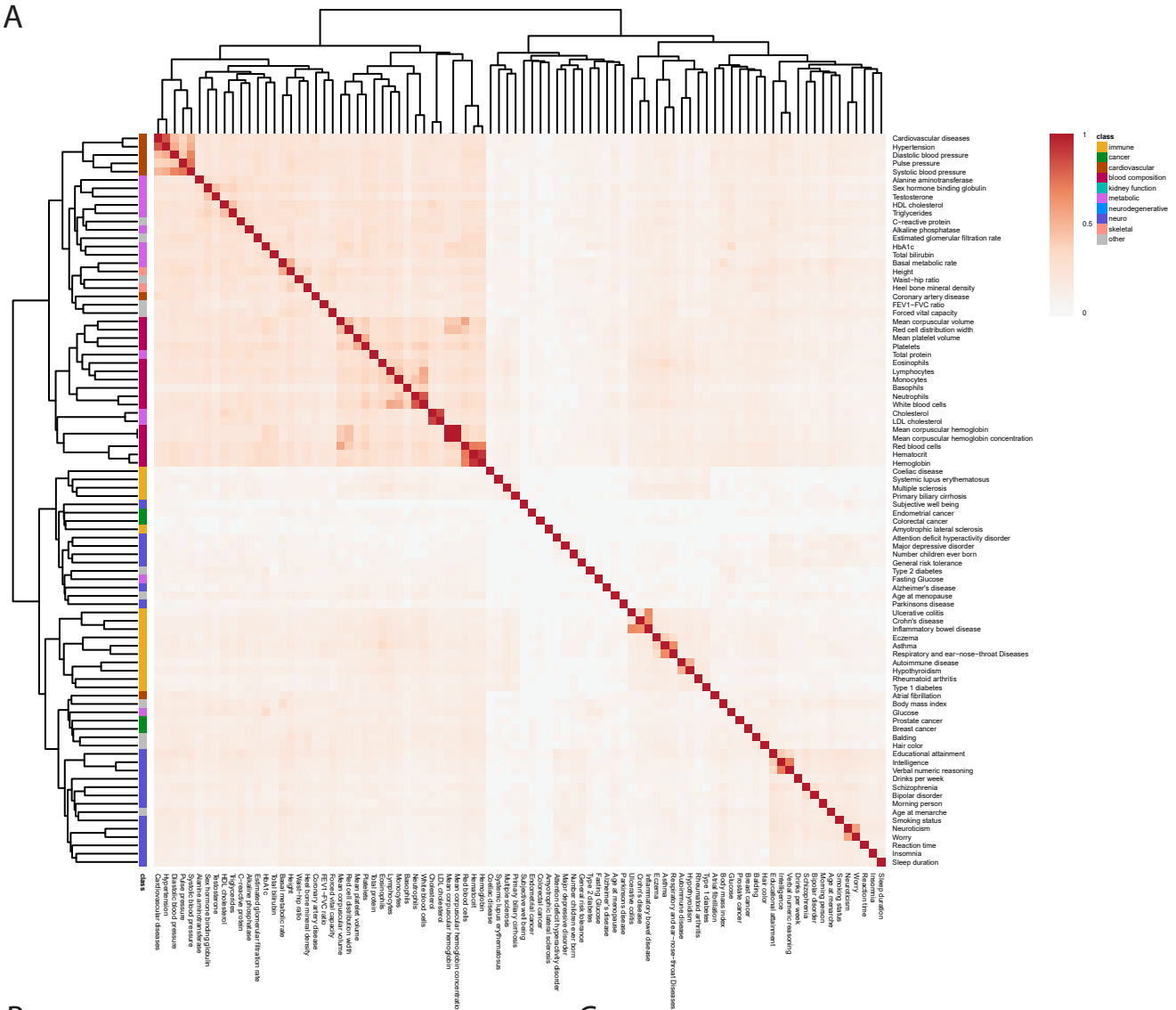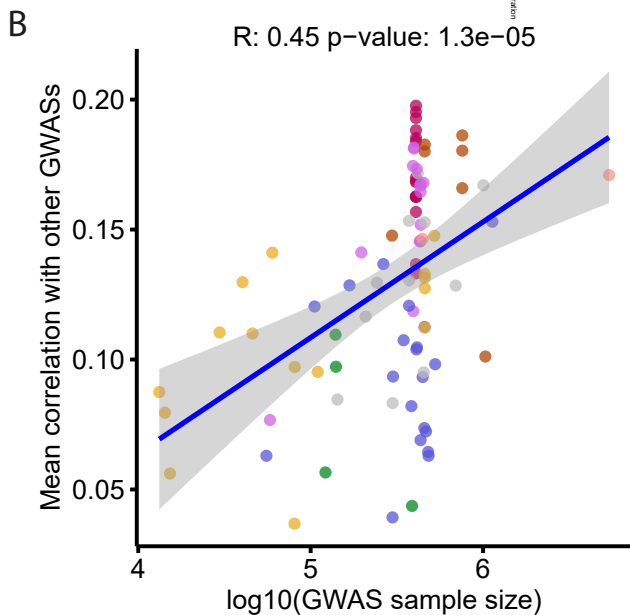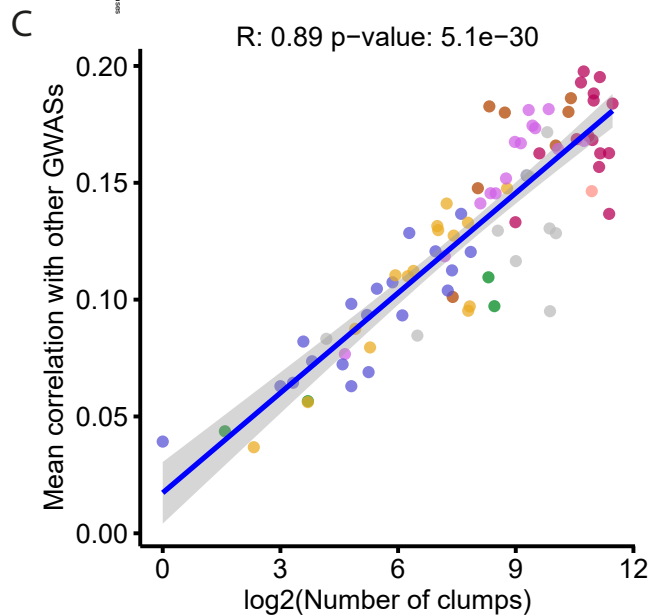

Supplement: Supplementary file 2 — Supplementary Information 2. [file 41598_2024_80670_MOESM2_ESM.pdf]
